# Lack of incremental prognostic value of triglyceride glucose index beyond coronary computed tomography angiography features for major events

**DOI:** 10.1038/s41598-024-77043-z

**Published:** 2024-10-27

**Authors:** Zengfa Huang, Ruiyao Tang, Yi Ding, Xi Wang, Xinyu Du, Wanpeng Wang, Zuoqin Li, Jianwei Xiao, Xiang Wang

**Affiliations:** 1 Department of Radiology, Tongji Medical College, The Central Hospital of Wuhan, Huazhong University of Science and Technology, 26 Shengli Avenue, Jiangan, Wuhan, 430014 Hubei China; 2 Department of Radiology, The Central Hospital of Wuhan Base, Hubei University of Medicine, Shiyan, 442000 Hubei China

**Keywords:** Triglyceride glucose index, Coronary computed tomography angiography, Coronary artery disease, Cardiology, Cardiovascular diseases

## Abstract

This study was aim to determine the prognostic value of triglyceride-glucose (TyG) index and coronary computed tomography angiography (CTA) features for major adverse cardiovascular events (MACE). In addition, we investigate the incremental prognostic value of TyG index beyond coronary CTA features in patients with suspected or known coronary artery disease (CAD). The present study ultimately includes 3528 patients who met the enrollment criteria. The TyG index was calculated based on measured levels of triglycerides and fasting blood glucose. Primary combined endpoint consisted of MACE, which defined as myocardial infraction (MI), all-cause mortality and stroke. Three multivariate Cox proportional hazard regression models were performed to assess the association between TyG index and MACE. C-statistic was performed to assess the discriminatory value of models. 212 (6.0%) patients developed MACE during a median follow-up of 50.4 months (IQR, 39.4–55.1). TyG index remained to be a significantly and independent risk factors for predicting MACE after adjusting by different models (clinical variables alone or plus coronary CTA features) in multivariable analysis. Both the addition of TyG index to clinical model plus Coronary Artery Disease Reporting and Data System (CAD-RADS) and to clinical model plus CAD-RADS 2.0 slightly but not significantly increased the C-statistic index (0.725 vs. 0.721, *p* = 0.223; 0.733 vs. 0.731, *p* = 0.505). TyG index was associated with an increased risk of MACE. However, no incremental prognostic benefit of TyG index over CAD-RADS or CAD-RADS 2.0 was detected for MACE in patients with suspected or known CAD.

## Introduction

Cardiovascular diseases (CVD) still remain the leading cause of global death and disability and coronary artery disease (CAD) is one of the most common cardiovascular disorders. In China, the mortality rate of CAD has been continuously increasing since 2012, with a significant increase in rural areas and surpassing urban since 2016 according to the data from China Health and Family Planning Statistics Yearbook (2022)^1^. These make early diagnosis and risk stratification of patients with CAD crucial for determining appropriate clinical management^2–4^. Coronary computed tomography angiography (CTA) has been regarded as a first-line noninvasive examination for the evaluation of CAD in the last decade^5^. The Coronary Artery Disease Reporting and Data System (CAD-RADS) which originally published in 2016 and updated in 2022 has archived accurately prediction value for major adverse cardiovascular events (MACE) and showed incremental prognostic values beyond coronary artery calcium (CAC) score and atherosclerotic cardiovascular disease risk score in patients with suspected CAD and stable chest pain^5,6^.

Recently, the triglyceride-glucose (TyG) index has been regarded as a reliable surrogate marker of insulin resistance. Accumulated studies have revealed that TyG index was positively associated with cardiovascular risk, including coronary artery stenosis, CAC score, obstructive CAD, symptomatic CAD and ischemic stroke^7–12^. Moreover, growing evidence has demonstrated that TyG index is a significant independent risk factor of morbidity and mortality of CVD^13–15^. However, little is known about the difference between TyG index and coronary CTA features, especially the CAD-RADS 2.0 classification for predicting future adverse events.

We therefore aim to determine the prognostic value of TyG index and coronary CTA features for MACE, and further to investigate the incremental prognostic value of TyG index beyond coronary CTA features in patients with suspected or known CAD.

## Methods

### Study population

The study population included patients who had undergone coronary CTA examinations for clinical reasons at our hospital from November 2018 and December 2020.

Inclusion criteria were patients aged 18 and above, with blood triglyceride and glucose tests before coronary CTA examination within one week. Exclusion criteria were patients who had revascularization treatments (percutaneous coronary intervention (PCI) or coronary artery bypass grafting, CABG), had a history of myocardial infraction (MI), missing coronary CTA images or reports (including stenosis information, plaque burden and Computed Tomography derived Fractional Flow Reserve, CT-FFR) and loss to follow-up.

The present study ultimately includes 3528 patients who met the enrollment criteria. The Central Hospital of Wuhan ethics committee granted the ethical approval and we confirmed that all methods were performed in accordance with the relevant guidelines and regulations.

## Data collection and definitions

Clinical data were collected from the medical records of the patients, including sex, age, smoking and drinking history, hypertension and diabetes history, and hypercholesterolemia history. Laboratory tests included triglycerides (TG), total cholesterol (TC), low-density lipoprotein cholesterol (LDL-C), high-density lipoprotein cholesterol (HDL-C), and fasting blood glucose (FBG). The TyG index was calculated according to the equation:

1 $$\:\text{T}\text{y}\text{G}\:\text{i}\text{n}\text{d}\text{e}\text{x}\:=\text{L}\text{n}\left[\frac{\text{T}\text{G}(\text{m}\text{g}/\text{d}\text{l})\times\:\text{F}\text{B}\text{G}(\text{m}\text{g}/\text{d}\text{l})}{2}\right]$$

This study classifies patients as low TyG index group (< 8.77) and high TyG index group (≥ 8.77) based on the median value of TyG index.

## Coronary CTA examination and analysis

All coronary CTA examinations were performed with prospectively or retrospectively ECG-triggered on dual-source CT scanner (Somatom Definition Flash, Siemens Medical Solutions, Forchheim, Germany). All the coronary CTA images were then analyzed automatically using an AI platform (Computer Aided Diagnosis of Coronary Artery, Version 6.0, Shukun technology, Beijing, China) for automatically generating standardized post-processing images and structured reporting. An 18-segment model of coronary anatomy was evaluated according to Society of Cardiovascular Computed Tomography guidelines^16^. Interpretation of the AI-based structured report for coronary CTA includes severity, extent, location of coronary stenosis, coronary plaque component, and CT FFR values. More detailed information on this AI-based coronary CTA platform was presented in previous work^17,18^ and National Medical Products Administration has approved this platform (No.20203210844 and No.20233210146).

CAD-RADS were evaluated for degree of stenosis using six categories as, CAD-RADS 0 (without stenosis), CAD-RADS 1 (1–24% stenosis), CAD-RADS 2 (25–49% stenosis), CAD-RADS 3 (50–69%), CAD-RADS 4 A (1 or 2 vessels with 70–99% stenosis), CAD-RADS 4B (3 vessels with 70–99% stenosis or left main ≥ 50% stenosis) and 5 (100% stenosis). We combined CAD-RADS 4B and CAD-RADS 5 as one composited classification in the present study.

Segment involvement score (SIS) were used to assess the plaque burden by categorizing as four groups: SIS 1 (≤ 2), SIS 2 (3–4), SIS 3 (5–7), and SIS 4 (≥ 8).

The CT-FFR classification was performed to evaluate the myocardial ischemia by categorized as two groups based on patients results: CT-FFR (> 0.80) and CT-FFR (≤ 0.80).

The CAD-RADS 2.0 contains CAD-RADS, SIS and CT-FFR classifications.

## Follow up

Primary combined endpoint consisted of MACE, which defined as MI, all-cause mortality and stroke. Follow-up procedures have been approved by our center’s institutional review board. Detail information of the follow-up methodology has been described previous work ^17^.

### Statistical analysis

Continuous variables are presented as median (interquartile range, IQR) or mean (standard deviation, SD) according to the data distribution. Categorical variables are described as frequency (percentage). The t-test, Wilcoxon or Kruskal-Wallis test was performed to compare group differences between continuous variables, and chi-square test was performed for comparing categorical variables. The variable, “person-years”, were calculated from the date of coronary CTA examination to either the date of MACE occurrence, or follow-up deadline. The Kaplan-Meier method was used to analyze the incidence of MACE and differences between/among groups with log-rank test. Three multivariate Cox proportional hazard regression models were performed to assess the association between TyG index and MACE by calculating the hazard ratio (HR) and 95% confidence interval (CI). Model 1 was adjusted by age, gender, smoke, hypertension, diabetes and hypercholesterolemia. Model 2 was further adjusted by CAD-RADS. Model 3 was further adjusted by SIS and CT-FFR. C-statistic was performed to assess the discriminatory value of models. CAD-RADS 4B and CAD-RADS 5 were combined as one composited classification as degrees had a relative low individual prevalence. All statistical analyses were carried out using SPSS (version 18, SPSS, Inc., Chicago, IL, USA), R statistical package (version 4.3.3, R foundation for Statistical Computing, Vienna, Austria) and MedCalc Statistical Software (version 16.8.4 Ostend, Belgium).

## Results

### Basic characteristics

A total of 3528 available patients were finally analyzed in the present study. The average age of the study patients was 61.1 (SD, 10.3) years old and 1597 (45.3%) were men. The median TyG index was 8.77 (IQR, 8.35–9.21). Table 1 showed the details basic characteristic of the study patients. According to the median TyG index, the study patients were divided into two groups (TyG index < 8.77 and high TyG index ≥ 8.77). Patients in the high TyG index group tend to more likely to be smokers, and have a history of hypertension and diabetes compared with patients in low TyG index group (all *p* < 0.05). TyG index were presented to be positively correlated with TC and LDL-C levels, whereas this correlation was inversely between TyG index and HDL-C level (all *p* < 0.05). In addition, patients in the high TyG index group were showed with higher degree of CAD-RADS and SIS classifications, and with lower CT-FFR values.

**Table 1:** Baseline characteristic and coronary CTA features of the study population.

Variables	Total (*N* = 3528)	TyG index < 8.77 (*N* = 1764)	TyG index ≥ 8.77 (*N* = 1764)	*P* Value
Age (years)	61.1 ± 10.3	61.8 ± 10.5	60.4 ± 10.1	0.211
Gender (Male, n, %)	1597 (45.3)	773 (43.8)	824 (46.7)	0.091
Smoke	812 (23.0)	372 (21.1)	440 (24.9)	0.007
Hypertension	1527 (43.3)	687 (38.9)	840 (47.6)	< 0.001
Diabetes	600 (17.0)	187 (10.6)	413 (23.4)	< 0.001
Hypercholesterolemia	913 (25.9)	866 (26.1)	47 (22.2)	0.225
TG, mmol/L	1.8 ± 1.5	1.8 ± 1.5	1.6 ± 0.9	0.117
TC, mmol/L	4.8 ± 1.1	4.6 ± 1.0	5.1 ± 1.1	0.016
HDL-C, mmol/L	1.3 ± 0.3	1.4 ± 0.4	1.2 ± 0.3	< 0.001
LDL-C, mmol/L	2.9 ± 1.0	2.8 ± 0.9	3.1 ± 1.2	0.006
CAD-RADS				< 0.001
0	1264 (35.8)	704 (39.9)	560 (31.7)	
1	802 (22.7)	390 (22.1)	412 (23.4)	
2	380 (35.3)	172 (9.8)	208 (11.8)	
3	515 (14.6)	236 (13.4)	279 (15.8)	
4 A	512 (14.5)	235 (13.3)	277 (15.7)	
4B&5	55 (1.6)	27 (1.5)	28 (1.6)	
SIS				< 0.001
≤ 2	2287 (64.8)	1228 (69.6)	1059 (60.0)	
3–4	514 (14.6)	216 (12.2)	298 (16.9)	
5–7	499 (14.1)	224 (12.7)	275 (15.6)	
≥ 8	228 (6.5)	96 (5.4)	132 (7.5)	
CT-FFR				< 0.001
> 0.80	2285 (80.9)	1479 (83.8)	1376 (78.0)	
≤ 0.80	673 (19.1)	285 (16.2)	388 (22.0)	

CTA = computed tomography angiography; TyG = triglyceride‑glucose; TG = triglyceride; TC = total cholesterol; HDL-C = high-density lipoprotein; LDL-C = low-density lipoprotein; CAD-RADS = coronary artery disease reporting and data system; SIS = segment involvement score; CT-FFR = computed tomography derived fractional flow reserve.

## Association between TyG index and MACE

**Fig. 1:**
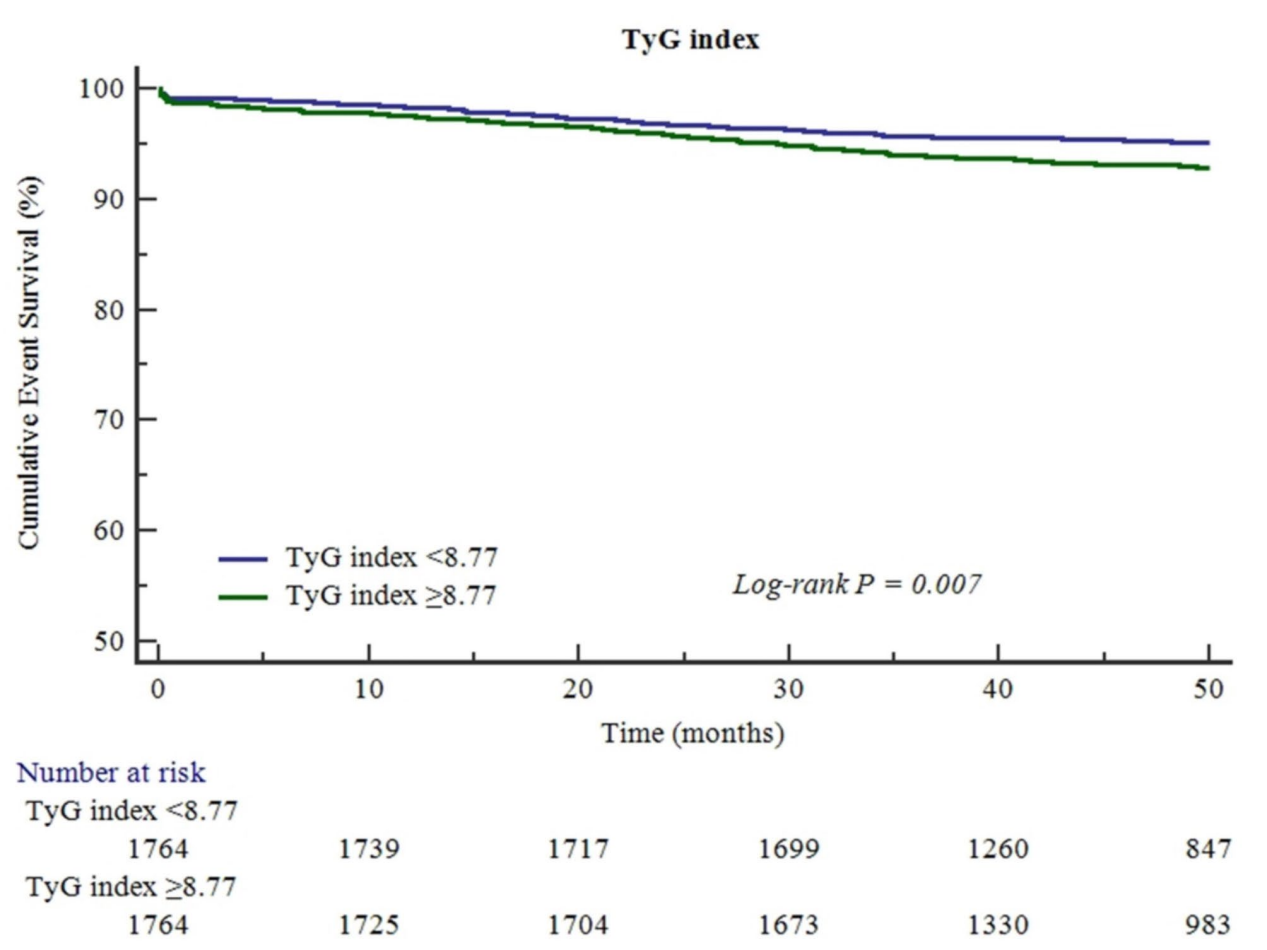
Association between TyG index and MACE. The number at risk of patients in TyG index < 8.77 group and TyG index ≥ 8.77 group decreased from 1764 at the start of follow-up to 847 and 983 at 50 months of follow-up, respectively. TyG, triglyceride‑glucose; MACE, major adverse cardiovascular events.

212 (6.0%) patients developed MACE during a median follow-up of 50.4 months (IQR, 39.4–55.1). The incidence of MACE was 1.25 (95%CI, 1.01–1.55) and 1.73 (95%CI, 1.45–2.06) per 100 person-years in low TyG index group and high TyG index group, respectively. Kaplan-Meier curves showed that patients in high TyG index group had a higher risk for MACE than that in low TyG index group (log-rank test, *p* = 0.007, Fig. 1).

**Fig. 2:**
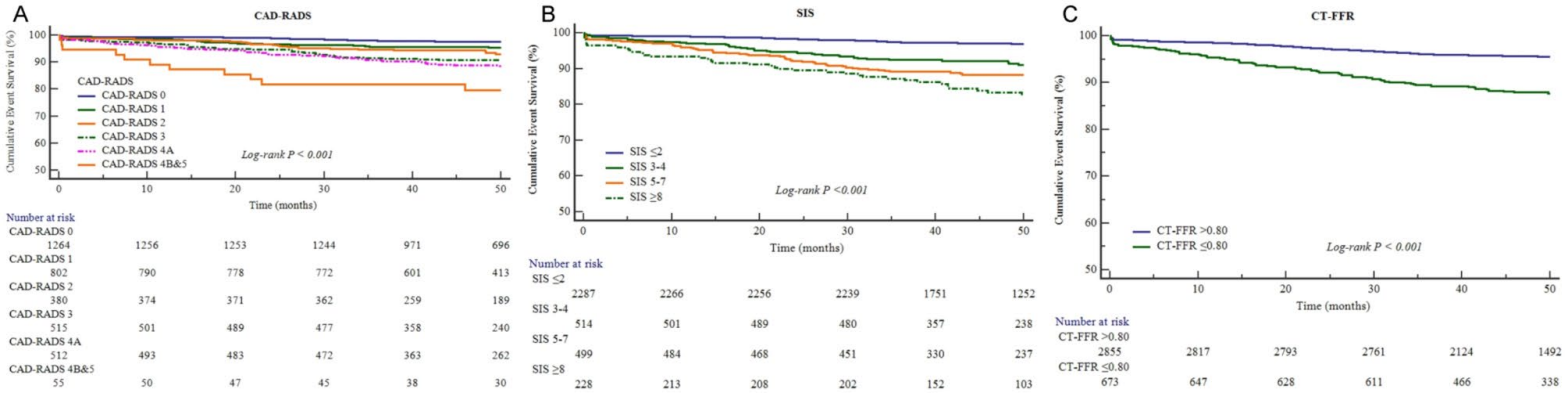
Association between CAD-RADS, SIS, CT-FFR and MACE. (**A**), The number at risk of patients in CAD-RADS 0, 1, 2, 3, 4 A and 4B&5 decreased from 1264, 802, 380, 515, 512, 55 at the start of follow-up to 696, 413, 189, 240, 262, 30 at 50 months of follow-up, respectively. (**B)**, The number at risk of patients in SIS ≤ 2, 3–4, 5–7 and ≥ 8 decreased from 2287, 514, 499, 228 at the start of follow-up to 1252, 238, 237, 103 at 50 months of follow-up, respectively. (**C**), The number at risk of patients in CT-FFR > 0.80 group and TyG index ≤ 0.80 group decreased from 2855 and 673 at the start of follow-up to 1492 and 338 at 50 months of follow-up, respectively. CAD-RADS = coronary artery disease reporting and data system; SIS = segment involvement score; CT-FFR = computed tomography derived fractional flow reserve; MACE, major adverse cardiovascular events.

Of the clinical risk factors, age (HR 1.06, 95% CI 1.05–1.08, *p* < 0.001), male (HR 1.48, 95% CI 1.13–1.94, *p* = 0.004), hypertension (HR 2.12, 95% CI 1.61–2.80, *p* < 0.001), diabetes (HR 2.16, 95% CI 1.61–2.90, *p* < 0.001) and TyG index (HR 1.46, 95% CI 1.12–1.92, *p* = 0.007) were significantly associated with MACE in univariable cox regression analysis. Of the coronary CTA features, gradual increases in risk were observed both for CAD stenosis burden and plaque burden: CAD-RADS 1 (HR 1.98, 95% CI 1.22–3.20, *p* = 0.006) and CAD-RADS 4B&5 (HR 11.04, 95% CI 5.76–21.17, *p* < 0.001); SIS 3–4 (HR 2.91, 95% CI 2.00–4.25, *p* < 0.001) and SIS ≥ 8 (HR 5.90, 95% CI 3.97–8.75, *p* < 0.001). In addition, CT-FFR ≤ 0.80 (HR 2.96, 95% CI 2.25–3.89, *p* < 0.001) also showed significantly associated with MACE (Table 2; Fig. 2). TyG index remained to be a significantly and independent risk factors for predicting MACE after adjusting by different models (clinical variables alone or plus coronary CTA features) in multivariable analysis (Table 3).

**Table 2:** Univariable Cox regression analysis for predicting MACE.

Variables	Univariate	
	HR (95% CI)	*P* Value
Age	1.06 (1.05–1.08)	< 0.001
Gender (male)	1.48 (1.13–1.94)	0.004
Smoke	1.27 (0.94–1.71)	0.127
Hypertension	2.12 (1.61–2.80)	< 0.001
Diabetes	2.16 (1.61–2.90)	< 0.001
Hypercholesterolemia	0.89 (0.78–1.01)	0.061
TyG **i**ndex	1.46 (1.12–1.92)	0.007
CAD-RADS		
0	1 (reference)	
1	1.98 (1.22–3.20)	0.006
2	2.74 (1.60–4.69)	< 0.001
3	4.30 (2.73–6.76)	< 0.001
4 A	5.00 (3.22–7.77)	< 0.001
4B&5	11.04 (5.76–21.17)	< 0.001
SIS		
≤ 2	1 (reference)	
3–4	2.91 (2.00–4.25)	< 0.001
5–7	4.17 (2.95–5.88)	< 0.001
≥ 8	5.90 (3.97–8.75)	< 0.001
CT-FFR		
> 0.80	1 (reference)	
≤ 0.80	2.96 (2.25–3.89)	< 0.001

MACE = major adverse cardiovascular events; TyG = triglyceride‑glucose; CAD-RADS = coronary artery disease reporting and data system; SIS = segment involvement score; CT-FFR = computed tomography derived fractional flow reserve.

**Table 3:** Multivariate Cox regression analysis for predicting MACE.

Variables	Model 1		Model 2		Model 3	
	HR (95% CI)	*P* Value	HR (95% CI)	*P* Value	HR (95% CI)	*P* Value
Age	1.06 (1.04–1.07)	< 0.001	1.05 (1.03–1.06)	< 0.001	1.04 (1.02–1.06)	< 0.001
Gender (male)	1.47 (1.07–2.02)	0.017	1.23 (0.92–1.66)	0.161	1.21 (0.87–1.67)	0.305
Smoke	1.06 (0.74–1.52)	0.738	1.06 (0.74–1.52)	0.738	1.06 (0.74–1.52)	0.738
Hypertension	1.42 (1.06–1.89)	0.018	1.27 (0.95–1.70)	0.107	1.21 (0.91–1.63)	0.195
Diabetes	1.70 (1.25–2.23)	0.001	1.50 (1.10–2.04)	0.010	1.39 (1.02–1.90)	0.036
Hypercholesterolemia	0.79 (0.55–1.12)	0.185	0.76 (0.53–1.08)	0.127	0.76 (0.53–1.08)	0.127
TyG **i**ndex	1.60 (1.18–2.17)	0.003	1.41 (1.05–1.90)	0.022	1.51 (1.11–2.05)	0.008
CAD-RADS						
0			1 (reference)		1 (reference)	
1			1.43 (0.88–2.34)	0.150	1.26 (0.75–2.10)	0.377
2			1.73 (1.00–3.01)	0.051	1.20 (0.64–2.24)	0.578
3			2.39 (1.48–3.86)	< 0.001	1.50 (0.83–2.72)	0.183
4 A			2.57 (1.59–4.14)	< 0.001	1.46 (0.77–2.75)	0.246
4B&5			4.45 (2.21–8.94)	< 0.001	2.17 (0.91–5.19)	0.080
SIS						
≤ 2					1 (reference)	
3–4					1.67 (1.05–2.65)	0.029
5–7					1.86 (1.13–3.04)	0.014
≥ 8					2.07 (1.15–3.74)	0.016
CT-FFR						
> 0.80					1 (reference)	
≤ 0.80					1.16 (0.79–1.69)	0.455

MACE = major adverse cardiovascular events; TyG = triglyceride‑glucose; CAD-RADS = coronary artery disease reporting and data system; SIS = segment involvement score; CT-FFR = computed tomography derived fractional flow reserve; Model 1 Adjusted by age, gender, smoke, hypertension, diabetes, hypercholesterolemia and TyG **i**ndex; Model 2 Adjusted by age, gender, smoke, hypertension, diabetes, hypercholesterolemia, TyG **i**ndex and CAD-RADS; Model 3 Adjusted by age, gender, smoke, hypertension, diabetes, hypercholesterolemia, TyG **i**ndex, CAD-RADS, SIS and CT-FFR.

### Incremental value of TyG index with coronary CTA feature for MACE

In order to further investigate the incremental prognostic value of TyG index for MACE, the C-statistic index were calculated and compared between different models (Table 4). The addition of CAD-RADS to clinical model showed borderline significance with increasing C-statistic index (0.721 vs. 0.703, *p* = 0.053). The addition of CAD-RADS 2.0 to clinical model plus CAD-RADS increased the C-statistic index significantly (0.731 vs. 0.721, *p* = 0.046). However, both the addition of TyG index to clinical model plus CAD-RADS and to clinical model plus CAD-RADS 2.0 slightly but not significantly increased the C-statistic index (0.725 vs. 0.721, *p* = 0.223; 0.733 vs. 0.731, *p* = 0.505).

**Table 4:** C-statistics for predicting MACE.

Models	C-statistic	*P* value
Clinical Model	0.703	
Clinical Model + CAD-RADS	0.721	0.053^*^
Clinical Model + CAD-RADS 2.0	0.731	0.046^$^
Clinical Model + CAD-RADS + TyG index	0.725	0.223^#^
Clinical Model + CAD-RADS 2.0 + TyG index	0.733	0.505^&^

TyG = triglyceride‑glucose; CAD-RADS = coronary artery disease reporting and data system; Clinical Model = age, gender, smoke, hypertension, diabetes and hypercholesterolemia; CAD-RADS 2.0 = CAD-RADS + SIS + CT-FFR; ^*^ Compared with Clinical Model; ^$^ Compared with Clinical Model + CAD-RADS; ^#^ Compared with Clinical Model + CAD-RADS; ^&^ Compared with Clinical Model + CAD-RADS 2.0.

## Discussion

The present study demonstrated that TyG index were independent predictors of MACE. However, adding TyG index to CAD-RADS or CAD-RADS 2.0 did not show incremental prognostic benefit in patients with suspected or known CAD.

### TyG index for predicting MACE

The present study found that a high TyG index was correlated with increasing risk of developing further MACE was supported by several retrospective and prospective studies^14,19^. The National Health and Nutrition Examination Survey III study revealed that a high TyG index was associated with a 21% higher subclinical myocardial injury risk in 6093 participants without CVD^19^. The recent Kailuan cohort study of 98,849 participants showed that a 2.08-fold risk of MI was observed in the higher TyG index (quartile 4) group compared with the low TyG group (quartile 1)^14^. In line with these studies, the current study of 3528 available patients who underwent coronary CTA examination with statistical power suggested that a high TyG index was associated with a 1.46-fold higher risk of MACE compared with low TyG index. After adjusted clinical risk factors and coronary CTA features, this significant association still persists.

### Coronary CTA features for predicting MACE

Coronary CTA has been demonstrated to accurately provide detailed information about the presence, severity and type of atherosclerotic plaque in the entire coronary artery tree. According to single most severe stenosis, CAD-RADS classification has been demonstrated with high predictive value of MACE^6,20–22^. However, the recent CORE320 trial showed that neither stenosis based classification or CAD-RADS classification provide an advantage beyond CAC score in 381 patients with stable angina^23^. The Western Denmark Heart Registry study also revealed that plaque burden was the main predictor of major CVD, defined as MI, stroke and mortality among 23,759 symptomatic patients^24^. The recently updated CAD-RADS 2.0 classification contains information of stenosis, plaque burden and myocardial ischemia, which offers more comprehensive information on atherosclerotic plaque burden and physiological significance. Both CAD-RADS and CAD-RADS 2.0 showed good prognostic abilities for MACE prediction. Furthermore, a borderline significance with better predictive value was observed in CAD-RADS classification than that of clinical model. In addition, the CAD-RADS 2.0 classification indicated the best prognostic abilities for MACE prediction than clinical model or clinical model plus CAD-RADS classification, which was consistent with previous studies^17,25,26^.

### Added value of TyG to coronary CTA features for predicting MACE

We have previously performed and validated the clinical usefulness of CAD-RADS and CAD-RADS 2.0 in prediction of MACE and all-cause mortality in patients with suspected or known CAD^17^. In the present study, we constructed predictive models for MACE among patients underwent coronary CTA examinations using clinical cardiovascular risk factors and coronary CTA features (CAD-RADS and CAD-RADS 2.0) and then further investigate the performance of combination models with adding TyG index. However, no improvement was detected in predicting MACE risk when further addition TyG index to CAD-RADS plus clinical model or CAD-RADS 2.0 plus clinical model during the follow-up time. Though the association between TyG index and poor outcomes has been revealed in accumulated evidence, only a few studies investigated the incremental prognostic value of TyG index to clinical model and showed inconsistent results. Jin et al.^27^ showed that adding TyG index to clinical model increased the prediction of cardiovascular outcomes (C-statistic index, 0.638 vs. 0.615, *p* = 0.002) in 1282 patients with type 2 diabetes mellitus and stable CAD in a nested case-control study. Moreover, Gao et al.^28^ revealed a significant improvement of risk evaluation after adding TyG index to clinical model in 1093 patients with coronary chronic total occlusion (AUC, 0.7244 vs. 0.629, *p* < 0.001). On the contrary, some studies presented no or partly improvement value in the prediction of CVD risk when add TyG index to clinical model. Barzegar et al.^29^ found that the addition of TyG index to the Framingham risk score did not improve the prediction of CVD risk in 7521 Iranians. In addition, Sanchez et al. showed an incremental predictive value of TyG index to Framingham risk score model among participants at intermediate 10-year cardiovascular risk (10–20%). However, this added predictive value of Framingham risk score model plus TyG index was not observed in low (< 10%) or high (> 20%) 10-year cardiovascular risk. However, to our knowledge, no published research has investigated the incremental prognostic value of TyG index to coronary CTA features, especially using the novel CAD-RADS 2.0 classification.

The study has some limitations. First, a causal relationship between TyG index and MACE risk could not be determined due to the observational nature of the present study. Thus, these findings need to be investigated with further prospective studies. Moreover, although adjustment of potential clinical risk factors of CAD was performed, the present study might still exist the possibility of the unmeasured or residual confounding. Second, there may be bias in the statistical results as treatments of the patients were not fully considered. Third, the patients in the present study were enrolled at a single hospital, and therefore the generalizability of these findings was limited.

## Conclusions

In conclusion, the present study showed that TyG index was associated with an increased risk of MACE. However, no incremental prognostic benefit of TyG index over CAD-RADS or CAD-RADS 2.0 was detected for MACE in patients with suspected or known CAD.

## Data Availability

The datasets generated during and analyzed during the current study are available from the corresponding author on reasonable request.
